# Age-Related Dynamics in the Conventional, Non-Conventional, and Bacteriological Characteristics of Fresh and Liquid-Stored Porcine Semen

**DOI:** 10.3390/ani15030377

**Published:** 2025-01-28

**Authors:** Eva Tvrdá, Ondřej Bučko, Michal Ďuračka, Anton Kováčik, Filip Benko, Miroslava Kačániová

**Affiliations:** 1 Institute of Biotechnology, Faculty of Biotechnology and Food Sciences, Slovak University of Agriculture in Nitra, Tr. A. Hlinku 2, 949 76 Nitra, Slovakia; evina.tvrda@gmail.com (E.T.); filip.benko@uniag.sk (F.B.); 2 Institute of Animal Husbandry, Faculty of Agrobiology and Food Resources, Slovak University of Agriculture in Nitra, Tr. A. Hlinku 2, 949 76 Nitra, Slovakia; ondrej.bucko@uniag.sk; 3 AgroBioTech Research Centre, Slovak University of Agriculture in Nitra, Tr. A. Hlinku 2, 949 76 Nitra, Slovakia; michal.duracka@uniag.sk; 4 Institute of Applied Biology, Faculty of Biotechnology and Food Sciences, Slovak University of Agriculture in Nitra, Tr. A. Hlinku 2, 949 76 Nitra, Slovakia; anton.kovacik@uniag.sk; 5 Institute of Horticulture, Faculty of Horticulture and Landscape Engineering, Slovak University of Agriculture in Nitra, Tr. A. Hlinku 2, 949 76 Nitra, Slovakia; 6 School of Medical and Health Sciences, University of Economics and Human Sciences in Warsaw, Okopowa 59, 010 43 Warsaw, Poland

**Keywords:** boars, age, semen quality, liquid storage, bacterial contamination, oxidative stress

## Abstract

This study strove to investigate the effect of boar age on conventional and non-conventional quality traits of fresh and liquid-stored semen. Sixty boars were allocated into 3 groups: 8–12 months (young); 24–36 months (adult); and 48–60 months (senior). Ejaculates were divided into two parts; the first one was assessed in native state while the second one was extended in the Androstar Plus extender containing gentamycin, stored at 5 °C and evaluated following 72 h. Young animals presented with a significantly lower sperm motility (*p* < 0.01), membrane and acrosome integrity (*p* < 0.0001), and mitochondrial activity (*p* < 0.0001) against adult boars. Significantly higher levels of free radicals and tumor necrosis factor alpha (*p* < 0.001), interleukin 1 and 6 (*p* < 0.0001) were found in young boars in comparison to adult boars. The assessment of liquid-stored semen revealed a significantly lower sperm motility, membrane, and acrosome integrity (*p* < 0.0001) in young boars when compared to adult boars. Moreover, *Clostridium difficile*, *Escherichia coli*, *Pseudomonas aeruginosa*, and *Rothia nasimurium* remained in liquid-stored semen obtained from young boars, while *Corynebacterium* sp. and *Escherichia coli* continued to be identified in samples collected from adult boars. In conclusion, age contributes to the overall quality of fresh as well as liquid-stored boar semen.

## Simple Summary

Intensive pig production by and large depends on high quality boar ejaculates to accomplish steady fertilization rates. To maximize the breeding process, males are introduced to the production as soon as possible. Nevertheless, this study suggests that young boars produce spermatozoa with less stable membranes and mitochondrial activity that are more susceptible to oxidative damage and bacteriospermia. Such predispositions may result in low sperm survival following liquid storage which may affect the success of artificial insemination. Similarly, a decline of sperm quality was observed in senior boars, that is affected by higher cell senescence and susceptibility to DNA damage, and hence to a lower ability of the sperm cell to successfully accomplish fertilization.

## 1. Introduction

Pig production is one of the most significant livestock sectors worldwide. Pork comprises more than 25% of total protein consumed globally and approximately 35% of meat production [1]. As the world population grows, and more people are expected to have higher incomes enabling more meat consumption, particularly in low- and middle-income countries, the demand for animal protein will likely increase, putting more pressure on intensive pig production [2].

Advances in contemporary pig production are closely linked to the implementation of assisted reproductive techniques, which have significantly contributed to a notable rise in the quantity and quality of pork products, including meat, fat, or skin [3]. Artificial insemination (AI) in particular has become a widespread choice for intensive pig breeding, as the majority of sows are being fertilized using AI exclusively in the largest swine production companies as well as smallholder production systems [3,4]. In comparison to natural breeding, AI offers several benefits, such as genetic improvement, the ability to choose from a wide selection of boars, the capacity to fertilize a large number of sows by using one semen sample, low disease transmission risks, fewer boars needed, and financial and time management resources [4,5]. Nevertheless, the success of AI relies on the quality of the semen specimen used for the procedure.

Boar semen quality may be affected by a variety of endogenous and exogenous factors, out of which the age of animals is among the most decisive ones. Previous studies indicate that the best reproductive performance is achieved at around 28–36 months, which is driven by high Leydig cell activity and a spike in testosterone levels [6,7,8,9]. Nevertheless, it is in the interest of intensive pig production to introduce stud males to the breeding program as soon as the animals reach sexual maturity, which oscillates at around 7–8 months. It has been previously reported that ejaculates collected from younger boars may present with higher numbers of spermatozoa with abnormal morphology [10], aberrant histone retention, and protamination [11], which may have a negative impact on the sperm survival if the semen samples are diluted and stored for later insemination. At the same time, a recent study of Li et al. [12] has identified differences in the seminal bacteriomes of boars of different ages, the overall load and diversity of which may have an impact on the resulting quality and oxidative balance of the ejaculates.

Semen quality is by and large assessed by a broad domain of traits, including macroscopic or microscopic parameters of the ejaculate, its biochemical composition, and cell vitality [13]. Most reports on the relationship between fertility and semen properties in boars have primarily considered sperm concentration, motility, or morphology as the principal attributes that may indirectly define fertility [8,9,10,11]. Nevertheless, it is evident now that the ejaculate is a heterogenous fluid comprising a complex network of molecules with multiple roles in sperm transport, motility enhancement, stimulation of capacitation or hyperactivation, maintenance of sperm viability, and protection of male gametes against stress [14].

In the meantime, it has been previously reported that most ejaculates collected from boars contain bacteria [15,16,17], which has been associated with low sperm motion activity, increased DNA fragmentation and cell death, morphological abnormalities, and premature acrosome reaction, all of which may compromise the suitability of the sample for artificial insemination [16,18]. Moreover, if such a contaminated sample is diluted in extenders rich in nutrients and at temperatures that may promote bacterial growth, the risk of transmitting a potential source of urogenital infection to sows represents a concerning issue for pig production [17,19].

While currently available studies on age-related semen differences in boars have been primarily limited to a relatively small set of sperm quality parameters, the impact of a complex set of variations in selected conventional and molecular indicators of sperm structural integrity and functional activity is still missing. In this study, we hypothesize that the age of the breeding boars may have impact on the semen quality beyond the most conventional sperm characteristics, some or all of which may have a decisive impact on the vitality and fertilization ability of male gametes. To verify this hypothesis, we employed a comprehensive comparative approach to investigate the ejaculates of the stud boars of three age groups. At the same time, we investigated age-dependent effects on the quality of liquid-stored boar semen, which could assist in the selection of a suitable age of boars for semen dilution and long-term storage in intensive pig breeding. Understanding the complexity of interactions amongst different constituents of semen that may be affected by the age of the animals may subsequently assist breeders to select the best strategy for semen collection, processing, dilution, storage, and dissemination in order to obtain the most desirable outcomes in terms of fertility and litter sizes in swine production.

## 2. Materials and Methods

### 2.1. Animals and Semen Collection

Semen samples were acquired from 60 breeding Duroc boars that had been housed at the pig breeding farm Terezov (Hlohovec, Slovakia) under the same production management. All animals had to accomplish a body condition score of 3. The boars were divided into three groups according to their age: (a) the young boar group comprised 20 males 8–12 months old (average weight of 262.15 ± 12.43 kg); (b) the adult boar group encompassed 20 males 24–36 months of age (average weight of 271.57 ± 14.65 kg); and (c) the senior boar group consisted of 20 males 48–60 months of age (average weight of 268.38 ± 15.97 kg). Boars were individually penned under a controlled environment temperature, fed with the same diets, and exposed to a total of 16 h of natural and artificial light. All animals were sexually active and under a weekly semen collection regimen. Prior to the onset of the experiments, the collection schedules of the animals included in the study were synchronized to avoid differences in the ejaculatory frequency. The animals were carefully handled in accordance with the ethical guidelines of the Slovak Animal Protection Regulation RD 377/12, conforming to European Union Regulation 2010/63.

Semen was collected during the spring period to exclude the seasonal effects on the sperm quality. Two samples were collected from each boar with a 3-day interval between the two collections. Overall, 120 samples were collected, with each group comprising 40 ejaculates. Ejaculates were collected by a qualified technician using the gloved-hand technique as follows: each boar was allowed to mount a collection dummy and attempt to copulate. The boar was then approached calmly and quietly from the rear. Preputial fluids were first evacuated by massaging the prepuce with double-gloved hands to prevent any eventual contamination of the ejaculate. The outer glove was removed and discarded, and the penis was allowed to thrust into the gloved hand. Following applying pressure, the penis became fully extended and ejaculation occurred. Sperm-rich fractions were collected into a sterile vessel and subsequently transported to the andrology laboratory in an isothermal vessel (37 °C) within 30 min. Each sample was divided into two identical parts—the first one was processed in its native state, and the second one was used for dilution and storage. Prior to the semen collection, precautionary measures were implemented to avoid external contamination of the specimens as outlined previously [17].

### 2.2. Native Semen Specimens

Each sample was diluted in PBS (phosphate buffered saline without calcium and magnesium; Sigma-Aldrich, St. Louis, MO, USA) using a ratio of 1:20 and subjected to the assessment of the sperm concentration and motility, the occurrence of leukocytes, membrane integrity, acrosome integrity, sperm apoptosis and necrosis, mitochondrial membrane potential, sperm DNA fragmentation index, and reactive oxygen species (ROS) production.

An aliquot of each specimen was centrifuged at 2300× *g* for 10 min to separate the cells from the seminal plasma. Seminal plasma aliquots were stored at −80 °C for subsequent analysis of the total antioxidant status (TAS), tumor necrosis factor alpha (TNF-α), interleukin 1 (IL-1), interleukin 6 (IL-6), C-reactive protein (CRP), lysozyme (LYZ), and general biochemistry. The cells were lysed with the RIPA buffer (Sigma-Aldrich, St. Louis, MO, USA) containing a protease inhibitor cocktail (Sigma-Aldrich, St. Louis, MO, USA) at 4 °C overnight. The samples were then centrifuged at 2300× g for 10 min, purified, and stored at −80 °C for the determination of oxidative damage to the proteins and lipids [20].

An aliquot of each native, undiluted specimen was transferred into a sterile Eppendorf tube and stored at −20 °C for bacteriology.

### 2.3. Semen Dilution and Storage

For the second experiment, we followed the standard procedure that is established by the pig farm to preserve ejaculates for later artificial insemination. Each semen sample was diluted with the Androstar Plus boar semen extender containing gentamycin (Minitüb, Tiefenbach, Germany) using a dilution ratio of 1:20. The diluted samples were stored at 5 °C for 72 h.

Following the storage period, each diluted semen sample was pre-warmed to 37 °C, an aliquot was transferred into a sterile Eppendorf tube, and stored at −20 °C for bacteriology [17]. Furthermore, each sample was subjected to the analysis of the sperm motility, membrane integrity, acrosome integrity, sperm apoptosis and necrosis, mitochondrial membrane potential, sperm DNA fragmentation index, and reactive oxygen species (ROS) production.

Similarly to native ejaculates, each liquid-stored specimen was centrifuged to remove the extender and to obtain the cells that served to prepare lysates for the determination of oxidative damage to the proteins and lipids [20].

### 2.4. Conventional Semen Quality Characteristics

Sperm concentration and motility were assessed with the HTM TOX IVOS II computer-assisted sperm analysis (CASA) system (Hamilton-Thorne Biosciences, Beverly, MA, USA). Each sample was loaded into the Makler counting chamber (Sefi Medical Instruments, Haifa, Israel), and at least 1000 spermatozoa were evaluated [17].

To assess the sperm viability, 1 × 10^6^ cells were stained with 10 μL CFDA (carboxyfluorescein diacetate; Sigma-Aldrich, St. Louis, MO, USA; 0.75 mg/mL in DMSO), 10 μL DAPI (4′,6-diamidino-2-phenylindole; Sigma-Aldrich, St. Louis, MO, USA; 1 μM in PBS), and at least 300 spermatozoa were examined under an epifluorescence microscope with a 40× magnification objective (Leica Microsystems, Wetzlar, Germany) as previously described [17].

For the acrosome integrity, 1 × 10^6^ cells were stained with 100 μL PNA (peanut agglutinin, FITC conjugate; Sigma-Aldrich, St. Louis, MO, USA; 10 μM in PBS), 10 μL DAPI, and at least 300 spermatozoa were analyzed with an epifluorescence microscope with 40× magnification as previously outlined [17].

The commercial Annexin-V-FLUOS Kit (Roche Applied Science, Basel, Switzerland) was used to assess phosphatidylserine dislocation indicative of apoptosis. The kit employs Annexin-V to stain early apoptotic spermatozoa and propidium iodide to distinguish necrotic spermatozoa. All samples adjusted to 1×10^6^ spermatozoa were stained with 1 μL Annexin-V and 1 μL propidium iodide, counterstained with 10 μL DAPI, transferred to a black 96-well plate, and the fluorescence intensity for each stain was quantified using the combined spectro-fluoro-luminometer (Glomax Multi^+^; Promega Corporation, Madison, WI, USA) [21].

The mitochondrial membrane potential was evaluated with the JC-1 Mitochondrial Membrane Potential Assay Kit (Cayman Chemical, Ann Arbor, MI, USA). Each specimen adjusted to 1 × 10^6^ spermatozoa was stained with the JC-1 dye and analyzed using the combined Glomax-Multi^+^ spectro-fluoro-luminometer as previously described [17].

Sperm DNA fragmentation index (%) was quantified with the Halomax commercial kit (Halotech DNA, Madrid, Spain) according to the instructions provided by the manufacturer. At least 300 processed spermatozoa were evaluated under an epifluorescence microscope with a 40× magnification objective as previously outlined [17].

### 2.5. Oxidative Profile

To determine global ROS levels in the samples, a luminol-based chemiluminescent method was used as previously published [17,20]. The light signal resulting from the interactions of luminol with endogenous as well as exogenous ROS was monitored in 15 one-minute-long cycles using the Glomax Multi^+^ spectro-fluoro-luminometer.

Total antioxidant status, representing a sum of all molecules with ROS-quenching or neutralizing abilities in the seminal plasma, was determined using the improved chemiluminescence antioxidant assay, which comprises horseradish peroxidase (Sigma-Aldrich; St. Louis, MO, USA), 4-iodophenol (Sigma-Aldrich; St. Louis, MO, USA), luminol, and hydrogen peroxide as a signal reagent and trolox (5–100 μmol/L; 6-hydroxy-2,5,7,8-tetramethylchroman-2-carboxylic acid; Sigma-Aldrich; St. Louis, MO, USA) as the standard [22]. The light signal was monitored in 10 consecutive one-minute-long cycles using the Glomax Multi^+^ spectro-fluoro-luminometer [20].

Levels of protein carbonyls, indicative of oxidative damage to the proteins in the cell lysates, were evaluated using the modified dinitrophenylhydrazine (DNPH) method as described previously [17,23]. Briefly, each sample was pre-treated with trichloroacetic acid (Sigma-Aldrich, St. Louis, MO, USA) and subsequently incubated with DNPH (Sigma-Aldrich, St. Louis, MO, USA). Unbound DNPH was washed out with ethanol/ethyl acetate (1:1; Sigma-Aldrich, St. Louis, MO, USA), and the cell pellets were resuspended in 6 M guanidine hydrochloride (Sigma-Aldrich, St. Louis, MO, USA) before being assessed at 360 nm on a UV–vis spectrophotometer (Cary Systems, Santa Clara, CA, USA).

Levels of malondialdehyde (MDA), indicative of the extent of lipid peroxidation in the cell lysates, were determined with the thiobarbituric acid-reactive substances (TBARS) method as previously outlined [20]. Briefly, each sample pre-treated with 5% SDS (sodium dodecyl sulfate; Sigma-Aldrich, St. Louis, MO, USA) was incubated in the presence of 0.53% thiobarbituric acid (Sigma-Aldrich, St. Louis, MO, USA) dissolved in 20% acetic acid (pH 3.5; Centralchem, Bratislava, Slovakia) in a water bath at 100 °C for 60 min. Following cooling down, the samples were transferred to a 96-well plate and assessed using the Glomax Multi^+^ spectro-fluoro-luminometer at a wavelength of 540 nm.

### 2.6. Immunological Profile

Quantification of leukocytes was performed with the Endtz test, which takes advantage of a detection solution consisting of 96% ethanol (Centralchem, Bratislava, Slovakia), benzidine (Sigma-Aldrich, St. Louis, MO, USA), sterile water, and 3% hydrogen peroxide (Sigma-Aldrich, St. Louis, MO, USA). Each specimen was mixed with the Endtz solution and evaluated under a bright-field microscope (×1000; Nikon ECLIPSE E100, Tokyo, Japan) as previously described [24].

Selected cytokines (TNF-α, IL-1, IL-6), inflammatory proteins (CRP), and antimicrobial peptides (LYZ) present in the seminal plasma were determined using commercially available ELISA kits (MyBioSource Inc., San Diego, CA, USA; cat. no. #MBS705977, #MBS2884704, #MBS701700, #MBS564088, and #MBS8807862, respectively). All tests were based on double-sandwich ELISA using a primary pre-coated monoclonal antibody and a secondary biotin-labeled polyclonal antibody. All measurements were performed as per the instructions of the manufacturer and executed with the help of the Glomax Multi^+^ spectro-fluoro-luminometer at 450 nm [17,24].

### 2.7. Seminal Plasma Biochemistry

Selected biochemical parameters of the seminal plasma, including calcium, magnesium, phosphorus, sodium, potassium, chloride, urea, uric acid, bilirubin, total proteins, albumin, cholesterol, and triglycerides, were measured using commercial kits purchased from Randox (Randox Laboratories, Crumlin, UK) on the fully automated clinical chemistry analyzer RX Monaco (Randox Laboratories, Crumlin, UK) [25].

### 2.8. Bacteriology

Bacterial isolation and identification followed a standard procedure that has been published earlier [17,20,24,25]. Briefly, each specimen was inoculated onto a selection of sterile agars (blood agar base no. 2; Gassner agar, NutriSelect^®^ basic; soybean casein digest agar; Merck, Darmstadt, Germany) and incubated under aerobic conditions at 36 ± 2 °C for 24 h. Subsequently, bacterial colonies were counted and transferred to fresh agars to obtain pure cultures, which were identified using the MALDI-TOF Biotyper mass spectrometer (Brucker Daltonics, Bremen, Germany). Bacterial identification was carried out with the Microflex LT instrument and flexControl software version 3.4. The spectra obtained by the Biotyper were linked with the MALDI Biotyper Bruker Taxonomy database (Bruker Daltonics, Bremen, Germany).

### 2.9. Statistics

The collected data were statistically processed with the GraphPad Prism program (version 10.4.0 for Mac; GraphPad Software Incorporated, La Jolla, CA, USA). The results are quoted as mean (±SD). The interpretation of the results was based on the value of the Pearson correlation coefficient (r): ±0.111–±0.333: weak correlation; ±0.334–±0.666: moderate correlation; ±0.667–±0.999: strong correlation. Significance levels were set at *p* < 0.05 (*), *p* < 0.01 (**), *p* < 0.001 (***), and *p* < 0.0001 (****).

## 3. Results

### 3.1. Conventional Semen Quality Characteristics

The comparative analysis revealed associations between the age of the animals and the conventional sperm characteristics, as evidenced by significant differences in the percentage of motile spermatozoa between all three pre-established groups, particularly when young boars were compared with adult (*p* < 0.01) and senior boars (*p* < 0.05) (Table 1). In comparison to adult and senior boars, specimens collected from young animals also presented with a significantly lower membrane integrity (*p* < 0.0001) and acrosome integrity (*p* < 0.0001). Significant differences in both parameters were also noted when adult and senior boars were compared against each other (*p* < 0.05). The highest occurrence of apoptotic and necrotic spermatozoa was detected in semen obtained from young boars, which was significantly higher, particularly in comparison with adult animals (*p* < 0.0001 in the case of apoptotic spermatozoa; *p* < 0.001 with respect to necrotic spermatozoa). Spermatozoa collected from adult boars presented with the highest mitochondrial membrane potential, which was significantly higher in comparison with young as well as senior boars (*p* < 0.0001). Moreover, the lowest degree of sperm DNA damage was recorded in adult boars, which was significantly different when compared to young (*p* < 0.0001) and senior boars (*p* < 0.001).

### 3.2. Oxidative Profile

In the case of ROS production, significantly higher levels of free radicals were recorded in young (*p* < 0.001) as well as senior boars (*p* < 0.05) when compared to adult animals (Table 2). Inversely, the highest TAS was found in adult boars, which was significantly higher in comparison to both young (*p* < 0.0001) and senior animals (*p* < 0.05). Significant differences were also detected amongst young and senior boars (*p* < 0.01). Correspondingly, protein oxidation was the most extensive in young boars and was significantly higher when compared to the adult (*p* < 0.001) and senior (*p* < 0.01) groups. At the same time, samples representing the young group presented with a significantly higher MDA content in comparison to the specimens obtained from the adult (*p* < 0.001) and senior (*p* < 0.01) groups.

### 3.3. Immunological Profile

The highest concentration of leukocytes was recorded in young boars, being significantly higher in comparison to adult as well as senior boars (*p* < 0.01) (Table 3). Young boars also presented with significantly higher TNF-α levels when compared to adult (*p* < 0.001) and senior animals (*p* < 0.05). The same observation was recorded in the case of IL-1 (*p* < 0.0001) as well as IL-6, although in this case, significant differences were observed only between the young and adult groups (*p* < 0.01). On the other hand, significantly higher CRP levels were found in young boars when compared to both adult (*p* < 0.001) as well as senior boars (*p* < 0.05).

At the same time, our results suggest that the higher the quality of the sample, the higher the content of lysozyme as a dominant antimicrobial protein. Significant differences in the LYZ concentration were observed between young and adult boars as well as among young and senior boars (*p* < 0.0001). Significantly different LYZ levels were also recorded amongst adult and senior animals (*p* < 0.001).

### 3.4. Seminal Plasma Biochemistry

In the case of meso- and microelements, the lowest calcium levels were recorded in young boars, which were significantly lower in comparison to adult (*p* < 0.01) and senior animals (*p* < 0.05) (Table 4). Similar differences were observed in the case of magnesium, significantly lower levels of which were found in young animals when compared to both adult and senior animals (*p* < 0.01). Whilst no significant differences were recorded in the case of phosphorus, significantly higher sodium levels were detected in specimens collected from young animals in comparison to their adult (*p* < 0.001) and senior counterparts (*p* < 0.05). On the other hand, significantly higher potassium levels were found in adult boars in comparison to young boars (*p* < 0.01). In the meantime, significantly higher chloride levels were recorded in young animals with significant differences against adult (*p* < 0.001) as well as senior animals (*p* < 0.05).

The highest urea levels were found in senior boars, being significantly different from young boars (*p* < 0.001). Significant differences in urea levels were also noted amongst young and adult boars (*p* < 0.01). Whilst no significant differences were found in the case of uric acid and bilirubin, significantly higher total protein levels were recorded in adult boars when compared to young boars (*p* < 0.0001). Significant differences in the levels of total proteins were also detected amongst young and senior boars (*p* < 0.05). Finally, no significant changes were observed in the case of the cholesterol and triglyceride levels amongst the pre-established groups.

### 3.5. Bacteriology

No significant differences in the total bacterial load as well as the quantity of coliform bacteria were found amongst the groups (Table 5); nevertheless, the samples differed in the bacterial diversity. Seven bacterial species were detected in the ejaculates collected from young boars, with *Escherichia coli* and *Pseudomonas aeruginosa* being the predominant species. Samples obtained from adult boars presented with 10 bacterial species, out of which *Bacillus licheniformis*, *Bacillus subtilis*, and *Corynebacterium* sp. were most frequently found in semen. Ten bacterial species were also identified in ejaculates collected from senior boars, with *Bacillus licheniformis*, *Bacillus subtilis*, *Escherichia coli*, and *Staphylococcus chromogenes* occurring most frequently in the samples.

### 3.6. Correlation Analysis

To fortify the comparative assessment, correlation analyses were performed in each pre-established age group to study age-based associations between the selected parameters of boar semen quality. According to Supplementary Table S1, increasing age of young animals was in a significant positive relation, particularly with the sperm motility (*p* < 0.05), membrane integrity (*p* < 0.01), and mitochondrial activity (*p* < 0.05), whilst a significant negative correlation was found between the increasing age of the boars and the occurrence of necrotic spermatozoa (*p* < 0.05), DNA fragmentation (*p* < 0.05), ROS levels (*p* < 0.05), as well as oxidative damage to the proteins and lipids (*p* < 0.01). While the levels of lysozyme (*p* < 0.05), calcium (*p* < 0.05), and total proteins (*p* < 0.01) were in a positive association with the age of young boars, negative correlations were found amongst the age of the animals, total bacterial load (*p* < 0.05), and the presence of coliform bacteria (*p* < 0.001).

In the case of adult boars, the correlation analysis revealed a significant negative association between the increasing age and total antioxidant capacity of the seminal plasma (*p* < 0.05), whilst significant positive correlations were found amongst the age of the boars, sodium, calcium, and phosphorus levels (*p* < 0.05) (Supplementary Table S2). Moreover, significant positive associations were also recorded between the increasing age, total bacterial load, and the presence of coliform bacteria (*p* < 0.05).

A notable shift in the associations between increasing age and semen quality parameters was observed in senior boars (Supplementary Table S3). The increasing age of the animals was found to be in a significant negative association with the sperm membrane integrity (*p* < 0.05), the occurrence of apoptotic spermatozoa (*p* < 0.05), DNA fragmentation (*p* < 0.05), and ROS levels (*p* < 0.05). Correspondingly, significant positive associations were observed amongst the increasing age of the boars, protein oxidation (*p* < 0.05), lipid peroxidation, as well as leukocyte concentration (*p* < 0.05) and interleukin 6 levels (*p* < 0.05). Moreover, the increasing age was significantly negatively correlated with the levels of calcium (*p* < 0.05), magnesium (*p* < 0.05), phosphorus (*p* < 0.05), total proteins (*p* < 0.05), and albumin (*p* < 0.05), whilst being in a significant positive association with the bacterial load (*p* < 0.05) and the presence of coliform bacteria (*p* < 0.05).

### 3.7. Liquid-Stored Semen Characteristics

Sperm motility assessment following 72 h of liquid storage revealed that the decline of sperm motility was significantly faster in young boars when compared to adult (*p* < 0.0001) as well as senior boars (*p* < 0.01) (Table 6). A significantly lower proportion of membrane-intact spermatozoa was found in the case of young boars in comparison to adult (*p* < 0.0001) or senior boars (*p* < 0.05). Correspondingly, a significantly lower acrosome integrity was recorded in liquid-stored spermatozoa obtained from young animals when compared to their adult or senior counterparts (*p* < 0.0001). At the same time, liquid-stored semen collected from young boars presented with a significantly higher proportion of apoptotic as well as necrotic spermatozoa, particularly in comparison with adult boars (*p* < 0.01). Following 72 h of liquid storage, the highest mitochondrial activity was detected in spermatozoa collected from adult boars, particularly in comparison to young boars (*p* < 0.001). At the same time, the lowest sperm DNA damage was recorded in liquid-stored semen from adult boars when compared to young animals (*p* < 0.01).

With respect to the oxidative balance of liquid-stored semen specimens, the highest ROS levels were observed in the case of young boars, which were significantly higher in comparison with adult (*p* < 0.0001) as well as senior boars (*p* < 0.001). Correspondingly, significantly higher levels of protein carbonyls (*p* < 0.001) as well as MDA (*p* < 0.01) were found in liquid-stored spermatozoa obtained from young boars, particularly in comparison with adult animals.

Bacteriological assessment of diluted semen stored for 72 h revealed a notable decrease of the total bacterial load as well as the quantity of coliform bacteria when compared to fresh, native semen. No significant differences were found amongst the pre-established groups in the case of the total bacterial load; nevertheless, a significantly decreased occurrence of coliform bacteria was recorded in liquid-stored specimens collected from adult boars when compared to young boars (*p* < 0.05). With respect to the variability of bacterial species present in the samples, *Clostridium difficile*, *Escherichia coli*, *Pseudomonas aeruginosa*, and *Rothia nasimurium* remained present in the ejaculates obtained from young boars while *Corynebacterium* sp. and *Escherichia coli* continued to be identified in liquid-stored samples collected from adult boars. In the meantime, *Corynebacterium* sp., *Escherichia coli*, *Pseudomonas aeruginosa* and *Rothia nasimurium* were retrieved from liquid-stored specimens obtained from senior boars.

## 4. Discussion

The role of age as an essential determinant of reproductive capacity in males has been a focal point of previous research in animals as well as in humans. Earlier studies have revealed that very young or senior age could have a significant impact on the sperm vitality and composition of ejaculates in bulls [26], rams [27], or dogs [28]. Correspondingly, previous reports have also indicated that age may affect morphometric and morphological attributes, chromatin integrity, and consequently in vitro fertilization ability of boar spermatozoa [10,11,29].

Conventional semen quantity and quality, by and large, depend on the individual attributes of each boar, which agrees with previous studies [11,30]. On the other hand, most young animals presented with a semen quality that rendered the samples as borderline suitable for liquid storage and eventual artificial insemination, particularly in terms of the sperm motility that, according to the pre-established cutoff values of the pig farm, should be higher than 60%. It is widely acknowledged that soon after young boars begin to produce semen, the average ejaculate volume is lower than it is in older animals. Previous studies agree that it is not until the 20th month of a boar’s life that the ejaculate volume stabilizes around an average of 300 mL per ejaculate, which is when the boar reaches its full sexual maturity [6,7,8,9,10]. A significant improvement in the sperm quality traits observed in adult boars agrees with Knecht et al. [8], Tsamadikis et al. [10], as well as Czubaszek et al. [11], who noted a significant improvement in the sperm quality in boars aged 24–36 months, comprising semen traits such as the semen volume, sperm concentration, viability, and morphology. Such differences may be explained by the fact that despite boars reaching sexual maturity, i.e., the ability to produce viable spermatozoa at approximately 6–8 months, the size of the testes, and additional glands, maturation of the parasympathetic system and scrotal circumference continue to grow to around 18 months of age [31,32]. Consequently, such adult boars will present with a significant increase in the quantity as well as quality of semen, which may also affect the conception rates and litter size [33]. On the other hand, it is well known that advancing male age is associated with a notable decrease in several key semen quality parameters, which may be caused by a plethora of factors, ranging from decreased antioxidant mechanisms and cell repair systems [34,35]; epididymal senescence [36], decreased numbers of germ cells and androgen levels [37], to alterations of the male reproductive anatomy, such as constriction of the seminiferous tubules or a decreased blood supply to the testes [38]. All such factors may ultimately contribute to a steady decrease in the quality of ejaculates, both in their fresh state as well as following liquid storage, as observed in this study.

Since the occurrence of bacteria in animal semen and their subsequent impact on the resulting semen quality as well as sperm survival during cold storage has become a pressing issue in animal reproduction science, a partial focus of this paper was to characterize age-dependent variations in the bacteriome of boar semen, whose impact on the resulting sperm quality depends on the diversity of bacterial species coupled with the quantity of bacteria present in the ejaculate, defined as the bacterial load [39]. In the case of the bacterial load, as opposed to studies on large farm animals [20,39,40], no significant variations in either the total bacterial count or the quantity of coliform bacteria were found among the pre-established groups. While it would seem plausible to speculate that in any highly specialized breed subjected to a greater selection and production pressure, the primary burden would be placed on the endogenous defense mechanisms predisposed to identify and oppose any potential pathogen [41], our results revealed that there were no differences among the studied age groups for the susceptibility of semen to contain bacteria. On the other hand, notable differences were observed in the bacterial diversity among the groups. *Acinetobacter lwoffii*, *Bacillus cereus*, *Clostridium difficile*, *Klebsiella pneumoniae*, or *Staphylococcus aureus* were not present in specimens collected from the adult group, which is subject to speculation since all animals included in this study were kept under identical conditions. Based on the collected data, we may speculate that the semen quality was impacted more by the bacterial diversity as opposed to the bacterial load, since the lowest sperm quality was found in young boars, which contained more potentially uropathogenic bacteria such as *Escherichia coli* [42], *Klebsiella pneumoniae* [43], or *Staphylococcus aureus* [44].

The specific molecular mechanisms by which bacteria affect sperm structural integrity and functional activity are complex. *Escherichia*, bacilli, or staphylococci frequently identified in boar ejaculates present with the ability to adhere to the cytoplasmic membrane on sperm surface and consequently interfere with the function of sperm receptors necessary for male gametes to interact with their surrounding [17,45,46,47,48,49,50,51]. In the case of staphylococci, sperm agglutination is frequently observed due to the secretion of the sperm agglutination factor (SAF) [47], whilst *E. coli* is frequently found attached to acrosomal receptors or the sperm flagellum, leading to sperm immobilization and subsequent death [46]. Our data concerning the membrane and acrosome integrity in relation to the presence of *Escherichia* and *Pseudomonas* may agree with previous studies [12,15,46], suggesting that their occurrence in semen is associated with a high proportion of spermatozoa with defects in the acrosome, sperm head, middle part, and flagellum, which, coupled with premature capacitation, may have negative consequences on the overall fertilization ability. The exact pathophysiology underlying such behavior has not yet been precisely understood; however, our data indicate a possible involvement of oxidative damage caused by ROS overproduction by the bacteria or by the release of proinflammatory cytokines as a response to bacteriospermia.

Another pathway of bacteria-induced spermatotoxicity is driven by bacterial toxins such as lipopolysaccharide (LPS), primarily secreted by G^−^ bacteria, and hemolysin as a primary product of *E. coli* or enterococci. The release of LPS subsequently triggers the toll-like receptor (TLR; specifically, TLR4), which in turn activates caspases and NF-κB, leading to a massive cytokine release, which, coupled with mitochondrial rupture, decreases the sperm motility [44]. On the other hand, hemolysin causes pore formation in the sperm membrane, which, coupled with ROS overproduction, leads to the membrane rupture and destabilization of the intracellular milieu. Such a phenomenon may lie behind a positive association between the presence of particularly coliform bacteria and a higher percentage of apoptotic and/or necrotic cells in the semen samples studied.

Besides a direct toxic effect that bacteria may exert on sperm physiology, several recent reports have shown that even commensal bacteria may affect the overall semen quality by competing with spermatozoa for nutrients provided by the seminal plasma [25,48]. Negative associations between the bacterial load and particularly biochemical components of the seminal plasma, such as calcium, magnesium, proteins, or albumin, strongly suggest that bacteria may deprive the seminal plasma of nutrients primarily designed to protect and nourish spermatozoa following ejaculation, which should be taken into consideration when designing semen extenders suitable for a long-term ex vivo preservation of spermatozoa.

In the meantime, a decline in the bacterial load as well as diversity was observed in adult as well as senior animals. Whilst the exact phenomenon to explain such changes has not been elucidated yet, we may speculate that better bacteriological outcomes may be linked to a higher maturity of the reproductive tract coupled with a higher degree of ejaculation frequency. It has been shown [8,41,42,43,44,45,46,47,48,49] that a more frequent and fully accomplished ejaculation may not only lead to improved sperm quality traits but also reduce the timeframe for the growth and accumulation of bacteria within the male urogenital tract. Furthermore, we may speculate that the inherent defense systems, including the adaptive immune system and the endogenous antioxidant mechanisms, may be far more mature and advanced in older animals; hence, the innate systems may have been more effective in the prevention and/or counteractivity of the negative effects bacteria may exhibit on male reproductive fluids and cells, as evidenced by our data collected from the oxidative and immunological profile of boar ejaculates.

Immunity is designed to oppose infection by releasing leukocytes to the source of inflammation. While under physiological circumstances white blood cells serve to remove potential pathogens as well as abnormal or dead spermatozoa, their overactivation may lead to phagocytosis of even viable male gametes [18,24,39]. Higher occurrence of leukocytes in ejaculates collected, particularly from young boars in this study, agrees with earlier reports [52,53] suggesting that leukocytospermia may negatively affect the stability of sperm membranes, even in otherwise healthy males. An activated immune system will also trigger the release of cytokines, which may exhibit detrimental effects on critical sperm biomacromolecules [54,55], leading to apoptosis and/or necrosis, as observed in this study. Within the heterogeneous group of pro-inflammatory molecules, TNF-α, IL-1, and IL-6, which are primarily secreted during infection, may promote the loss of membrane integrity, phosphatidylserine translocation, and subsequent cell death [56], which corresponds with their increasing levels proportionately to a decreased semen quality as observed in this study as well as in previous reports on stud animals [18,24,39]. High cytokine levels have also been reported to be correlated with oxidative stress [57], mitochondrial dysfunction, and the loss of sperm motion activity [18,39,58], which is also corroborated by our data. What is more, Barranco et al. [55] speculate that specific boar seminal plasma cytokines may contribute to sperm structural and metabolic changes during semen preservation, either in liquid or frozen state, which is also strongly supported by our data collected on the sperm survival in liquid-stored state. On the other hand, lysozyme is gaining increased attention as an important representative of the antibacterial properties of seminal plasma. Our data strongly agree with previous reports [44,59] suggesting that lysozyme levels increase in parallel with the maturity of the male reproductive system of boars and that the protein may be at least partially responsible for the absence of *Staphylococcus aureus* or a significant decrease of *Escherichia coli* isolates in semen obtained from older animals.

In parallel with inflammation, supraphysiologic ROS levels released by spermatozoa, leukocytes, aerobic, or facultative aerobic bacteria may lead to the onset and/or progression of oxidative damage to spermatozoa [60]. Proportionately to elevated ROS levels, significantly increased damage to the sperm proteins and lipids was detected, particularly in young animals. Oxidative insults to the membrane lipid bilayer may subsequently compromise the semipermeable properties of the sperm surface [60,61]. Our data also agree with previously published studies suggesting that cell death could be intricately involved in the promotion of ROS-inflicted sperm DNA fragmentation [18,39,62]. Correspondingly, a higher occurrence of spermatozoa with altered membranes and aberrant mitochondrial activity was accompanied by sperm chromatin disintegration, which has been frequently observed in suboptimal boar semen [11,63,64].

Another important outcome of this study lies in changes observed in the seminal plasma of the pre-established groups. Since the knowledge on the roles of the seminal plasma biochemistry on male fertility is limited, we are currently left to hypothesize how the plasma components may impact the resulting sperm vitality and ex vivo survival. Data collected from the biochemical analysis revealed lower levels of phosphorus, calcium, and magnesium in the seminal plasma of young boars. It is well known that all three ions are involved in the regulation of sperm architecture and physiology, such as ATP synthesis, sperm motility, capacitation, and the acrosome reaction, all of which are vital for a successful fertilization [65,66,67]. Increased levels of all ions detected in older animals suggest their important roles as sperm vitality promoters and fortify their positive correlations with sperm motility reported in earlier studies on boar semen [25,68].

The composition and levels of seminal plasma proteins have also been shown to play important roles in semen quality, acting in the prevention of hyperosmolality and oxidative stress and acting as oxidizable substrates for spermatozoa [69]. In this study, higher levels of seminal plasma proteins and albumin were found in adults and senior boars in comparison to young boars. Although it was previously hypothesized that the total seminal plasma protein content may not be a fully reliable indicator of low semen quality [70], previous reports have suggested that low seminal plasma protein levels were correlated with a decreased sperm motion activity [25,47,71]. In the meantime, albumin is a major seminal plasma protein involved in molecular transport, sperm maturation, acrosome reaction, and sperm–oocyte fusion [72,73], which has been associated with optimal semen quality and longevity under ex vivo conditions [71,74].

The outcomes of the post-liquid storage sperm vitality reveal a significant decrease in all quality parameters, particularly in the samples collected from young boars. Given the initial sperm quality that was lower in younger animals in comparison to adult or senior animals, it seems that the samples were already compromised and hence more vulnerable to ex vivo stress. A more notable decrease of membrane and acrosome integrity following the storage period may be connected to a higher incidence of morphological abnormalities that have been reported to be more frequent in previous studies [10,29,31]. At the same time, it has been suggested that the ex vivo environment may cause additional stress on the mitochondrial function, leading to the loss of ATP, crucial for sperm movement, and subsequent ROS overproduction by defective spermatozoa [29,75]. A lower sperm survival in young animals may also be connected to a higher degree of immature spermatozoa that are particularly vulnerable to iatrogenic, thermal, and oxidative stress. Such factors may place additional burden on male gametes, which may subsequently respond with mitochondrial dysfunction, increased ROS release, and a higher incidence of DNA breakage as observed in this study.

What is more concerning is the observation that gentamicin was not efficient enough to eradicate all bacteria present in native ejaculates. Furthermore, *Escherichia coli* or *Pseudomonas aeruginosa* were found in liquid-stored samples, despite the fact that gentamicin is more effective against G^−^ bacteria [75], which agrees with previous reports [75,76,77] suggesting that bacteria routinely found in boar ejaculates may present with resistance to gentamicin. Such observations may result in a deduction that while currently available boar semen extenders may comply with the 90/429/EEC European Council Directive laying down the animal health requirements applicable to intra-community trade in and imports of semen of domestic animals of the porcine species, their efficiency to prevent bacterial contamination of liquid-stored semen may be limited.

## 5. Conclusions

Since swine production directly relies on the quality of semen, attention must be paid to all the factors that may potentially put the sperm viability in jeopardy during the collection, processing, and storage of ejaculates. Summarizing the outcomes of this study, several key findings may be conveyed. Semen is a heterogenous bodily fluid holding an intricate and complex network of interactions that may ultimately affect sperm quality. Besides conventional semen quality characteristics such as sperm concentration, motility, or membrane integrity, advanced markers of sperm structural integrity and functional activity, including mitochondrial function or DNA fragmentation, may offer a broader view of ex vivo sperm behavior and survival. An important aspect lies in the importance of the seminal bacteriome, which behaves as a dynamic entity, the load and diversity of which may significantly impact the semen properties. In addition, oxidative stress and inflammation seem to act as major contributors to substandard semen quality, which is why we may suggest the implementation of their basic assessment, particularly in fresh ejaculates, prior to their further processing and dissemination. On the other hand, the seminal plasma chemistry seems to play an important role in the protection of sperm vitality, which is why increased attention should be devoted to its potential use in porcine reproductive sciences.

Given the primary aim of this study, we may emphasize the existence of differences among boars of different ages in terms of particularly the stability of membranous structures and mitochondrial activity, susceptibility to oxidative stress, and bacterial contamination. Such predispositions may lead to lower sperm survival following liquid storage, which may have a significant impact on the success of artificial insemination. If ejaculates collected from young or older boars are used in the breeding process, precautionary measures should be implemented during semen processing, such as sperm selection techniques, physical removal of bacteria, and administration of antioxidants or alternative antibacterial supplements to the semen extenders.

## Figures and Tables

**Table 1:** Age-related variations in conventional semen quality characteristics in breeding boars.

Semen Quality Parameter	Young Boars (*n* = 40)	Adult Boars (*n* = 40)	Senior Boars (*n* = 40)
Volume [mL]	147.00 ± 13.64	136.80 ± 7.19	134.20 ± 10.03
Concentration [×10^6^/mL]	419.20 ± 30.01	792.70 ± 36.13 *^Y^	723.60 ± 18.15
Sperm motility [%]	60.67 ± 8.83	83.00 ± 3.89 **^Y^	77.20 ± 4.26 *^Y^
Membrane integrity [%]	70.33 ± 2.75	89.00 ± 1.42 ****^Y^	83.00 ± 4.52 ****^Y;^*^A^
Acrosome integrity [%]	69.83 ± 3.62	90.00 ± 2.10 ****^Y^	84.60 ± 4.22 ****^Y;^*^A^
Apoptotic spermatozoa [%]	17.50 ± 1.39	9.60 ± 1.74 ****^Y^	13.60 ± 1.74 **^Y;^**^A^
Necrotic spermatozoa [%]	11.67 ± 0.74	7.20 ± 0.73 ***^Y^	9.80 ± 1.52 *^Y^
Mitochondrial membrane potential [JC1 green/red ratio]	0.75 ± 0.03	0.89 ± 0.03 ****^Y^	0.87 ± 0.02 ****^Y^
Sperm DNA fragmentation index [%]	13.83 ± 0.95	8.60 ± 1.02 ****^Y^	9.80 ± 1.16 ***^Y^

*—*p* < 0.05; **—*p* < 0.01; ***—*p* < 0.001; ****—*p* < 0.0001. ^Y^—vs. young boars; ^A^—vs. adult boars.

**Table 2:** Age-related variations in seminal oxidative characteristics in breeding boars.

Semen Quality Parameter	Young Boars (*n* = 40)	Adult Boars (*n* = 40)	Senior Boars (*n* = 40)
Reactive oxygen species production [RLU/s/10^6^ sperm]	10.33 ± 1.12	7.20 ± 1.17 ***^Y^	9.40 ± 1.36 *^A^
Total antioxidant capacity [eq. μmol Trolox eq./g prot.]	15.67 ± 2.21	25.00 ± 2.76 ****^Y^	21.00 ± 1.79 **^Y;^*^A^
Protein carbonyl levels [nmol PC/mg prot.]	8.16 ± 0.69	4.80 ± 0.60 ****^Y^	5.80 ± 0.40 **^Y^
Malondialdehyde levels [μmol MDA/g prot.]	2.26 ± 0.21	1.42 ± 0.31 ****^Y^	1.74 ± 0.18 **^Y^

*—*p* < 0.05; **—*p* < 0.01; ***—*p* < 0.001; ****—*p* < 0.0001. ^Y^—vs. young boars; ^A^—vs. adult boars.

**Table 3:** Age-related variations in seminal immunological characteristics in breeding boars.

Semen Quality Parameter	Young Boars (*n* = 40)	Adult Boars (*n* = 40)	Senior Boars (*n* = 40)
Leukocyte concentration [×10^6^/mL]	10.40 ± 1.06	2.72 ± 0.71 **^Y^	3.07 ± 0.51 **^Y^
Tumor necrosis factor α levels [pg/mg prot.]	1.97 ± 0.25	1.36 ± 0.24 ***^Y^	1.58 ± 1.17 *^Y^
Interleukin 1 levels [pg/mg prot.]	177.00 ± 12.18	125.60 ± 8.23 ****^Y^	135.60 ± 7.92 ****^Y^
Interleukin 6 levels [pg/mg prot.]	0.19 ± 0.02	0.14 ± 0.03 **^Y^	0.18 ± 0.03
C-reactive protein levels [mg/g prot.]	0.96 ± 0.05	0.64 ± 0.12 ***^Y^	0.78 ± 0.16 *^Y^
Lysozyme levels [μg/g prot.]	89.50 ± 8.02	201.20 ± 7.88 ****^Y^	177.40 ± 10.56 ****^Y;^***^A^

*—*p* < 0.05; **—*p* < 0.01; ***—*p* < 0.001; ****—*p* < 0.0001. ^Y^—vs. young boars; ^A^—vs. adult boars.

**Table 4:** Age-related variations in the seminal plasma biochemistry in breeding boars.

Semen Quality Parameter	Young Boars (*n* = 40)	Adult Boars (*n* = 40)	Senior Boars (*n* = 40)
Calcium levels [mmol/L]	0.67 ± 0.16	0.94 ± 0.07 **^Y^	0.87 ± 0.16 *^Y^
Magnesium levels [mmol/L]	3.63 ± 0.64	4.39 ± 0.05 *^Y^	4.24 ± 0.17 *^Y^
Phosphorus levels [mmol/L]	0.31 ± 0.04	0.46 ± 0.03	0.45 ± 0.05
Sodium levels [mmol/L]	119.80 ± 6.86	98.38 ± 5.42 ***^Y^	106.40 ± 10.33 *^Y^
Potassium levels [mmol/L]	14.34 ± 0.87	16.40 ± 1.36 **^Y^	15.35 ± 0.87
Chloride levels [mmol/L]	109.00 ± 8.53	85.66 ± 5.14 ***^Y^	95.94 ± 9.93 *^Y^
Urea levels [μmol/L]	0.11 ± 0.02	1.75 ± 0.15 **^Y^	2.15 ± 0.40 ***^Y^
Uric acid levels [μmol/L]	0.44 ± 0.07	0.48 ± 0.07	0.54 ± 0.06
Bilirubin levels [μmol/L]	7.03 ± 0.86	8.09 ± 0.65	10.25 ± 1.36
Total protein levels [g/L]	10.02 ± 1.66	23.84 ± 1.75 ****^Y^	17.52 ± 2.51 *^Y^
Albumin levels [g/L]	1.67 ± 0.21	3.64 ± 0.57 ***^Y^	2.27 ± 0.16 *^A^
Cholesterol levels [mmol/L]	0.32 ± 0.03	0.41 ± 0.09	0.33 ± 0.06
Triglyceride levels [mmol/L]	0.25 ± 0.02	0.31 ± 0.07	0.26 ± 0.06

*—*p* < 0.05; **—*p* < 0.01; ***—*p* < 0.001; ****—*p* < 0.0001. ^Y^—vs. young boars; ^A^—vs. adult boars.

**Table 5:** Age-related variations in the bacterial profiles of fresh, native semen in breeding boars.

Semen Quality Parameter	Young Boars (*n* = 40)	Adult Boars (*n* = 40)	Senior Boars (*n* = 40)
Total bacterial load [logCFU/mL]	4.23 ± 0.39	4.17 ± 0.49	4.18 ± 0.61
Coliform bacteria [logCFU/mL]	3.61 ± 0.71	2.31 ± 0.58	2.43 ± 0.88
Identified bacterial species and sample positivity (percentage of samples with the identified bacterial species)	*Acinetobacter lwoffii * (20.00%) *Clostridium difficile * (40.00%) *Escherichia coli * (80.00%) *Klebsiella pneumoniae * (20.00%) *Pseudomonas aeruginosa* (65.00%) *Rothia nasimurium * (20.00%) *Staphylococcus aureus* (20.00%)	*Aerococcus viridans * (15.00%) *Bacillus licheniformis * (40.00%) *Bacillus subtilis * (50.00%) *Corynebacterium* sp. (40.00%) *Escherichia coli * (25.00%) *Proteus vulgaris * (15.00%) *Pseudomonas aeruginosa* (10.00%) *Pseudomonas putida * (25.00%) *Staphylococcus chromogenes* (20.00%) *Staphylococcus simulans* (20.00%)	*Bacillus cereus * (20.00%) *Bacillus licheniformis * (60.00%) *Bacillus subtilis * (40.00%) *Corynebacterium* sp. (20.00%) *Escherichia coli * (40.00%) *Proteus vulgaris * (20.00%) *Pseudomonas aeruginosa* (20.00%) *Rothia nasimurium * (20.00%) *Staphylococcus chromogenes* (40.00%) *Staphylococcus simulans* (25.00%)

**Table 6:** Age-related variations in selected quality parameters of liquid-stored boar semen.

Semen Quality Parameter	Young Boars (*n* = 40)	Adult Boars (*n* = 40)	Senior Boars (*n* = 40)
Sperm motility [%]	48.20 ± 6.68	70.90 ± 3.29 ****^Y^	64.60 ± 8.40 **^Y^
Membrane integrity [%]	57.20 ± 6.36	79.40 ± 4.49 ****^Y^	73.40 ± 7.81 *^Y^
Acrosome integrity [%]	60.40 ± 4.22	81.80 ± 2.10 ****^Y^	76.80 ± 3.32 ****^Y^
Apoptotic spermatozoa [%]	26.20 ± 7.19	15.60 ± 2.42 **^Y^	18.40 ± 3.56 *^Y^
Necrotic spermatozoa [%]	19.00 ± 5.89	10.20 ± 2.04 **^Y^	13.20 ± 3.54
Mitochondrial membrane potential [JC1 green/red ratio]	0.55 ± 0.06	0.80 ± 0.05 ***^Y^	0.73 ± 0.09 **^Y^
Sperm DNA fragmentation index [%]	24.00 ± 6.12	14.20 ± 0.98 **^Y^	17.40 ± 3.45
Reactive oxygen species production [RLU/s/10^6^ sperm]	25.80 ± 4.71	14.20 ± 2.04 ****^Y^	17.00 ± 1.79 ***^Y^
Protein carbonyl levels [nmol PC/mg prot.]	14.80 ± 1.92	6.00 ± 0.89 ***^Y^	8.20 ± 0.75 **^Y^
Malondialdehyde levels [μmol MDA/g prot.]	3.96 ± 0.90	2.14 ± 0.59 **^Y^	2.48 ± 0.56 **^Y^
Total bacterial load [logCFU/mL]	1.17 ± 0.36	0.64 ± 0.19	0.71 ± 0.13
Coliform bacteria [logCFU/mL]	0.48 ± 0.10	0.22 ± 0.09 *^Y^	0.38 ± 0.07
Identified bacterial species and sample positivity (percentage of samples with the identified bacterial species)	*Clostridium difficile * (10.00%) *Escherichia coli * (10.00%) *Pseudomonas aeruginosa * (10.00%) *Rothia nasimurium * (10.00%)	*Corynebacterium* sp. (5.00%) *Escherichia coli * (5.00%)	*Corynebacterium* sp. (10.00%) *Escherichia coli * (5.00%) *Pseudomonas aeruginosa* (5.00%) *Rothia nasimurium * (5.00%)

*—*p* < 0.05; **—*p* < 0.01; ***—*p* < 0.001; ****—*p* < 0.0001. ^Y^—vs. young boars.

## Data Availability

The data presented in this study are available on request from the corresponding author.

## Supplementary Materials

The following supporting information can be downloaded at: https://www.mdpi.com/article/10.3390/ani15030377/s1, Table S1: Age-related correlations in conventional, non-conventional, oxidative, immunological and bacteriological semen quality characteristics in young boars. Table S2: Age-related correlations in conventional, non-conventional, oxidative, immunological and bacteriological semen quality characteristics in adult boars. Table S3: Age-related correlations in conventional, non-conventional, oxidative, immunological and bacteriological semen quality characteristics in senior boars.
