# Supplementary material for: Age-Related Dynamics in the Conventional, Non-Conventional, and Bacteriological Characteristics of Fresh and Liquid-Stored Porcine Semen
Source: Animals (Basel). 2025 Jan 28;15(3):377. doi: 10.3390/ani15030377 (PMC11815876; doi:10.3390/ani15030377)
Supplement: Supplementary file 1 [file animals-15-00377-s001.zip › animals-3429551-supplementary.pdf]

Supplementary Table 1. Age-related correlations in conventional, non-conventional, oxidative, immunological and bacteriological semen quality characteristics in young boars.

	Age	Volume	Conc.	Motility	Memb. I.	Acros. I.	Apopt.	Necrot.	Mit. Act.	DNA Fr.	ROS	TAC	Prot. Ox.	LPO	Leukoc	TNFα	IL-1	IL-6	CRP	Lysoz.	Ca	Mg	P	Na	K	Cl	Urea	Uric Ac.	Bilir.	Tot. Pr.	Albumin	Chol.	Trigl.	CFU	Colif.
Age	1																																		
Volume	-0.190	1																																	
Conc.	0.339*	0.433	1																																
Motility	0.425*	-0.153	0.430*	1																															
Memb. I.	0.572**	-0.195	-0.444*	0.577**	1																														
Acros. I.	0.201	-0.202	-0.368*	0.458*	0.662**	1																													
Apopt.	-0.166	0.183	0.513*	-0.789**	-0.737***	-0.449*	1																												
Necrot.	-0.464*	0.230	0.591*	-0.617*	-0.715***	-0.490*	0.807***	1																											
Mit. Act.	0.379*	0.142	-0.360*	-0.624*	0.442**	-0.371*	-0.580*	-0.749***	1																										
DNA Fr.	-0.392*	0.143	-0.561*	-0.377*	-0.538**	-0.390*	0.833***	0.611**	-0.358*	1																									
ROS	-0.392*	0.103	0.374*	-0.713**	-0.682**	-0.859***	0.544*	0.741**	-0.452*	0.833***	1																								
TAC	0.315*	-0.122	-0.470*	0.511**	0.510*	0.469*	-0.653**	-0.539**	0.527*	-0.669**	-0.818***	1																							
Prot. Ox.	-0.602**	0.231	-0.302*	-0.456*	-0.494*	-0.355*	0.262	0.408*	-0.727*	0.725**	0.731**	-0.585**	1																						
LPO	-0.524**	0.131	-0.334*	-0.658**	-0.685**	-0.654**	0.761**	0.390*	-0.621*	0.568*	0.940***	0.464*	0.786**	1																					
Leukoc	-0.238	0.206	0.212	-0.513*	-0.459*	-0.470*	0.731**	0.533**	-0.391	0.719**	0.872***	-0.403*	0.668**	0.718***	1																				
TNFα	-0.187	0.186	0.391*	-0.644**	-0.832***	-0.215	0.434*	0.634**	-0.664**	0.671**	0.371*	0.651**	0.726**	0.317	0.616**	1																			
IL-1	-0.123	0.121	0.665**	-0.600**	-0.799**	-0.649**	0.328	0.380*	-0.362*	0.411*	0.567*	-0.391*	0.700**	0.554**	0.541**	0.504**	1																		
IL-6	-0.532*	0.208	0.585**	-0.474*	-0.307*	-0.771***	0.505*	0.738**	-0.409*	0.450*	0.842***	-0.368*	0.746**	0.631**	0.548**	0.498**	0.773***	1																	
CRP	-0.131	0.079	0.261	-0.346	-0.461**	-0.831***	0.397*	0.797***	-0.403*	0.379*	0.874***	-0.397*	0.741**	0.710***	0.575**	0.432*	0.727***	0.851	1																
Lysoz.	0.365*	-0.187	0.388*	0.455**	0.487**	0.365*	-0.065	-0.236	0.396*	-0.807**	-0.375*	0.368*	-0.667**	-0.427*	-0.585**	-0.450*	-0.778***	-0.888	-0.487*	1															
Ca	0.555**	-0.378*	-0.482*	0.379*	0.809***	0.406*	-0.062	-0.479*	0.422*	-0.101	-0.055	0.255	-0.112	-0.248	-0.433	-0.689**	-0.472*	-0.023	-0.319	0.078	1														
Mg	0.417*	-0.320*	-0.496*	0.420*	0.652**	0.519*	-0.543*	-0.782***	0.571**	-0.416	-0.219	0.319	-0.178	-0.194	-0.505*	-0.511*	-0.425	-0.152	-0.219	0.132	0.669**	1													
P	0.403*	-0.302	0.390*	0.235	0.123	0.474*	-0.457*	-0.318	0.463*	-0.626	-0.398*	0.398*	-0.408*	-0.187	-0.002	-0.308	0.320	0.276	-0.242	0.031	0.317	0.283	1												
Na	-0.411	0.317*	0.565**	0.275	0.402*	0.123	-0.379*	-0.316	0.257	-0.346	-0.284	0.284	-0.165	-0.401	-0.449*	-0. 244	-0.035	-0.010	0.085	0.256	-0.161	-0.153	-0.204	1											
K	-0.395	-0.357*	-0.184	0.246	0.177	0.806**	-0.249	-0.423	-0.149	-0.380	-0.250	0.350	-0.348	-0.185	0.005	-0.019	-0.077	-0.079	-0.172	-0.259	-0.254	0.275	0.284	-0.587*	1										
Cl	-0.377	0.391*	0.441*	0.333	0.398*	0.066	-0.361	-0.456*	0.415	-0.309	-0.277	0.277	-0.461*	-0.420	-0.436*	0.241	0.125	0.021	-0.032	0.050	-0.217	-0.245	-0.290	0.968**	-0.512*	1									
Urea	-0.274	0.414*	0.283	0.235	-0.301	-0.204	-0.383*	-0.187	-0.150	-0.305	-0.279	0.490*	0.191	-0.278	-0.454*	0.415	0.299	-0.075	0.434*	-0.083	-0.485	-0.159	-0.446*	0.818***	-0.518*	0.895**	1								
Uric Ac.	-0.283	0.345*	0.259	0.266	-0.076	0.093	-0.329	-0.156	-0.354	-0.320	-0.355	0.535*	-0.209	0.032	-0.473*	0.264	0.173	-0.093	0.270	-0.117	-0.061	-0.141	-0.113	0.604*	-0.064	0.675*	0.827**	1							
Bilir.	0.213	-0.060	0.198	0.220	0.224	0.064	-0.103	-0.286	-0.136	-0.111	-0.309	0.309	-0.194	-0.174	0.062	0.255	0.181	0.056	-0.293	-0.054	0.064	0.071	-0.326	0.151	-0.139	0.257	0.032	-0.299	1						
Tot. Pr.	0.617**	0.698**	0.293	0.540*	0.433*	0.343	-0.774**	-0.419*	0.377	-0.611**	-0.180	0.801***	-0.259	-0.317*	-0.446*	0.299	-0.197	-0.1415	0.047	0.044	0.037	0.288	-0.153	0.840	-0.676**	0.882**	0.978***	0.728**	0.010	1					
Albumin	0.283	0.424*	-0.337*	0.402*	0.565*	0.361*	-0.451*	-0.597**	0.596**	-0.490*	-0.466*	0.686**	-0.474*	-0.163	-0.413	-0.474	-0.433*	-0.226	-0.090	0.035	0.511*	0.222	0.527**	-0.891**	0.293	0.328*	0.380**	0.369	-0.059	0.816***	1				
Chol.	0.403*	0.276	-0.482*	0.566*	0.457*	0.519**	-0.431*	-0.107	0.854**	-0.121	-0.223	0.523*	-0.298	0.286	-0.094	-0.143	-0.182	0.034	0.044	-0.169	-0.056	-0.126	-0.291	0.556*	0.142	0.565*	0.469	0.745**	-0.020	0.348	-0.403*	1			
Trigl.	0.272	0.298	0.372*	0.540*	0.433*	0.343*	-0.774**	-0.699**	0.077	-0.611*	-0.301	0.201	-0.259	0.317	0.043	0.085	0.197	-0.041	0.047	0.044	-0.037	-0.188	-0.553*	0.840**	-0.676**	0.882**	0.978***	0.728**	0.010	0.089	-0.416*	0.348*	1		
CFU	-0.365*	0.375	0.492*	-0.543*	-0.490*	-0.377*	0.477*	0.522*	-0.401*	0.443*	0.490*	-0.461*	0.389*	0.444*	0.568**	0.613**	0.607**	0.548	0.784**	0.310	-0.478*	0.383*	0.487*	-0.426*	-0.356*	-0.331	0.073	-0.038	-0.278	-0.114	-0.373*	-0.620***	0.114	1	
Colif.	-0.848***	0.367	0.663**	-0.747**	-0.519**	-0.510*	0.504**	0.532*	-0.543**	0.495*	0.510**	-0.526**	0.722***	0.661**	0.655**	0.701***	0.738***	-0.660	0.779**	0.406	-0.520**	0.361*	0.358*	-0.401	0.374**	-0.551**	-0.495*	-0.051	-0.703***	-0.490*	-0.488**	-0.172	-0.490*	0.802***	1
	Age	Volume	Conc.	Motility	Memb. I.	Acros. I.	Apopt.	Necrot.	Mit. Act.	DNA Fr.	ROS	TAC	Prot. Ox.	LPO	Leukoc	TNFα	IL-1	IL-6	CRP	Lysoz.	Ca	Mg	P	Na	K	Cl	Urea	Uric Ac.	Bilir.	Tot. Pr.	Albumin	Chol.	Trigl.	CFU	Colif.

The interpretation of the results was based on the value of the Pearson's correlation coefficient: 0.111–0.333: weak correlation; 0.334–0.666: moderate correlation; 0.667–0.999: strong correlation. * – $p < 0.05$; ** – $p < 0.01$; *** – $p < 0.001$. Conc.: sperm concentration; Memb. I.: membrane intergity; Acros. I.: acrosome integrity; Mit. Act.: mitochondrial activity; DNA Fr.: sperm DNA fragmentation; ROS: reactive oxygen species; TAC: total antioxidant status; Prot. Ox.: protein oxidation (protein carbonyls); LPO – lipid peroxidation; Leukoc.: leukocyte levels; TNFα – tumor necrosis factor alpha; IL-1: interleikin 1; IL-6: interleukin 6; CRP: C-reactive protein; Lysoz.: lysozyme; Ca: calcium; Mg: magnesium; P: phosphorus; Na: sodium; K: potassium; Cl: chloride; Uric. Ac.: uric acid; Bilir.: bilirubin; Tot. Pr.: total protein; Chol: cholesterol; Trigl.: triglycerides; CFU – colony forming units (total bacterial load); Colif.: coliform bacteria.

Supplementary Table 2. Age-related correlations in conventional, non-conventional, oxidative, immunological and bacteriological semen quality characteristics in adult boars.

	Age	Volume	Conc.	Motility	Memb. I.	Acros. I.	Apopt.	Necrot.	Mit. Act.	DNA Fr.	ROS	TAC	Prot. Ox.	LPO	Leukoc	TNFα	IL-1	IL-6
Age	1																	
Volume	1.000	1																
Conc.	-0.097	0.637**	1															
Motility	0.022	0.726**	0.887***	1														
Memb. I.	-0.123	0.413*	0.465*	0.701***	1													
Acros. I.	-0.174	0.208	0.417*	0.515**	0.566**	1												
Apopt.	0.094	-0.501**	-0.369*	-0.643**	-0.706***	-0.516**	1											
Necrot.	0.233	-0.347	-0.820***	-0.809***	-0.707**	-0.377*	0.853***	1										
Mit. Act.	-0.134	0.492**	0.691**	0.795***	0.416*	0.396*	-0.585**	-0.555**	1									
DNA Fr.	0.152	-0.147	0.468*	-0.580**	0.416*	-0.496*	0.652**	0.680***	0.438*	1								
ROS	0.128	0.458**	0.507**	-0.541*	-0.643**	-0.857***	0.551**	0.662***	0.537**	0.701***	1							
TAC	-0.399*	-0.262	-0.699***	0.623**	0.462*	0.871***	-0.566**	-0.547**	-0.485*	-0.413*	-0.860***	1						
Prot. Ox.	0.098	0.153	0.396*	-0.474*	-0.354*	-0.938***	0.674**	0.607***	0.572**	0.419*	0.879***	-0.771***	1					
LPO	0.236	0.795**	0.429*	-0.538**	-0.890***	-0.851***	0.617**	0.738***	0.775***	0.406*	0.889***	-0.605**	0.522**	1				
Leukoc	0.389*	0.158	0.568*	-0.423*	-0.455*	-0.456*	0.518*	0.656***	0.498*	0.590**	0.766***	-0.298	0.510**	0.667**	1			
TNFα	0.314	0.789***	0.384*	-0.458*	-0.527**	-0.414*	0.850***	0.337	0.714***	0.471*	0.525**	-0.120	0.386*	0.608**	0.636**	1		
IL-1	0.224	-0.251	0.381	-0.715***	-0.475*	-0.449*	0.365*	0.941***	0.338*	0.614**	0.529**	-0.555**	0.389*	0.593**	0.838***	0.513**	1	
IL-6	0.028	-0.287	0.464*	-0.443*	-0.410*	-0.602**	0.407*	0.833***	0.482**	0.612**	0.619**	-0.536**	0.369*	0.364*	0.629***	0.783***	0.741***	1
CRP	0.233	0.056	0.406*	-0.358*	-0.589**	-0.917***	0.554**	0.576**	0.333	0.523**	0.629**	-0.907***	-0.896	0.384*	0.663**	0.683**	0.447**	0.398*
Lysoz.	0.194	-0.355	0.201	0.369*	0.405*	0.862***	-0.791***	-0.661**	0.483*	-0.389*	-0.918***	0.580**	-0.964***	-0.527**	-0.540**	-0.558*	-0.576**	-0.344*
Ca	0.531*	-0.460*	-0.323*	0.871***	0.502*	0.589**	-0.582**	-0.962***	0.552**	-0.606**	-0.276	0.529**	-0.192	-0.073	-0.654**	0.177	-0.056	-0.161
Mg	0.130	-0.483**	-0.494*	0.923***	0.439*	0.438*	-0.771***	-0.931***	0.586**	-0.609**	0.013	0.456**	-0.059	-0.291	-0.370*	0.133	-0.098	-0.152
P	0.432*	-0.904***	-0.494**	0.768**	0.202	0.316	-0.713***	0.297	0.524*	-0.024	-0.243	0.191	-0.397	-0.006	-0.362**	-0.169	-0.155	-0.157
Na	0.418*	0.489*	0.689**	0.460*	0.561**	0.127	-0.420*	-0.536**	0.526*	-0.023	-0.120	-0.017	-0.019	-0.120	-0.380*	-0.150	-0.712	-0.110
K	0.266	0.099	-0.226	0.211	0.663**	0.087	-0.278	0.073	0.313	-0.241	0.205	-0.128	0.151	0.052	-0.351*	-0.139	-0.85	-0.029
Cl	0.257	0.527**	0.718**	0.589**	0.815**	0.193	-0.623**	-0.656**	0.587**	-0.350*	-0.009	-0.016	0.123	-0.020	-0.221	0.169	-0.-53	-0.216
Urea	0.078	-0.265	0.264	-0.141	0.558*	0.185	-0.171	-0.294	-0.243	0.090	-0.134	0.072	-0.357*	-0.195	-0.297	-0.168	-0.048	-0.278
Uric Ac.	0.228	0.075	0.669**	0.623**	0.502*	0.275	0.049	-0.704***	0.131	0.051	-0.432**	0.448*	-0.501**	0.036	-0.218	-0.399*	-0.061	0.073
Bilir.	-0.120	-0.730***	-0.888***	0.392	-0.045	-0.209	0.095	0.009	-0.036	0.201	0.035	0.420*	-0.303	-0.102	-0.065	-0.403*	-0.096	-0.190
Tot. Pr.	-0.090	-0.314	-0.840***	0.630**	0.536**	0.243	-0.407*	-0.846***	0.390*	-0.659**	-0.423*	0.348*	0.660**	-0.232	-0.061	0.044	-0.058	-0.161
Albumin	-0.132	-0.192	-0.164	0.493*	0.550**	0.443*	-0.932***	-0.531**	0.491**	-0.718***	-0.470*	0.501**	-0.684**	-0.264	-0.059	0.241	-0.275	-0.275
Chol.	0.230	0.081	0.375*	0.472*	0.788***	0.777**	-0.167	-0.358**	0.102	-0.318*	-0.362*	0.088	-0.476*	-0.377*	0.059	-0.291	-0.237	0.201
Trigl.	0.123	-0.033	0.247	0.011	0.490*	0.850***	-0.757***	0.058	0.086	-0.647***	-0.650*	0.075	-0.325*	-0.044*	-0.155	0.022	-0.091	0.093
CFU	0.386*	0.481*	0.502**	-0.519*	-0.846***	-0.522*	0.499**	-0.474*	-0.422**	0.644**	0.660**	-0.434*	0.530**	0.575**	0.796**	0.526**	0.670**	0.686**
Colif.	0.336*	0.431*	0.447*	-0.550**	-0.897***	-0.560**	0.527***	0.521**	-0.433**	0.731***	0.880***	-0.667**	0.560**	0.662**	0.828***	0.599**	0.749***	0.719***
	Age	Volume	Conc.	Motility	Memb. I.	Acros. I.	Apopt.	Necrot.	Mit. Act.	DNA Fr.	ROS	TAC	Prot. Ox.	LPO	Leukoc	TNFα	IL-1	IL-6

	CRP	Lysoz.	Ca	Mg	P	Na	K	Cl	Urea	Uric Ac.	Bilir.	Tot. Pr.	Albumin	Chol.	Trigl.	CFU	Colif.
CRP	1																
Lysoz.	-0.774***	1															
Ca	-0.155	0.229	1														
Mg	-0.039	0.251	0.925***	1													
P	0.080	0.117	0.428*	0.606	1												
Na	-0.214	0.316	-0.206	-0.285	-0.249	1											
K	-0.063	0.277	0.188	-0.169	-0.160	-0.701***	1										
Cl	-0.296	0.188	-0.298	-0.155	-0.183	0.969***	-0.527**	1									
Urea	-0.080	0.183	-0.134	-0.007	0.340*	0.678**	-0.042	0.055	1								
Uric Ac.	-0.194	0.320*	0.317*	0.277	0.089	0.007	0.154	0.051	0.006	1							
Bilir.	-0.159	0.309*	0.355*	0.242	0.347*	-0.427*	0.063	-0.108	0.010	-0.167	1						
Tot. Pr.	-0.051	0.684**	0.915***	0.717**	0.257	-0.079	0.562**	-0.094	-0.175	-0.130	0.525**	1					
Albumin	-0.161	0.766**	0.461*	0.709**	0.513**	-0.160	0.431*	-0.094	0.237	0.090	0.238	0.777**	1				
Chol.	-0.139	0.394*	-0.256	-0.237	0.182	-0.106	0.525*	-0.050	-0.134	0.174	-0.057	0.033	0.075	1			
Trigl.	-0.152	0.865***	0.049	0.230	0.255	-0.211	-0.121	-0.060	0.038	0.295	-0.042	0.048	0.087	0.651**	1		
CFU	-0.612**	-0.874***	-0.592**	-0.540**	-0.452*	-0.445*	-0.828***	-0.895***	0.356*	-0.079	-0.190	-0.848***	-0.518**	-0.538**	-0.267	1	
Colif.	-0.709***	-0.877***	-0.880*	-0.860***	-0.500*	-0.688**	-0.744**	-0.627**	0.410*	-0.618***	-0.333	-0.408*	-0.301	-0.920***	-0.559**	0.821***	1
	CRP	Lysoz.	Ca	Mg	P	Na	K	Cl	Urea	Uric Ac.	Bilir.	Tot. Pr.	Albumin	Chol.	Trigl.	CFU	Colif.

The interpretation of the results was based on the value of the Pearson's correlation coefficient: 0.111–0.333: weak correlation; 0.334–0.666: moderate correlation; 0.667–0.999: strong correlation. * – $p < 0.05$; ** – $p < 0.01$; *** – $p < 0.001$. Conc.: sperm concentration; Memb. I.: membrane intergity; Acros. I.: acrosome integrity; Mit. Act.: mitochondrial activity; DNA Fr.: sperm DNA fragmentation; ROS: reactive oxygen species; TAC: total antioxidant status; Prot. Ox.: protein oxidation (protein carbonyls); LPO – lipid peroxidation; Leukoc.: leukocyte levels; TNFα – tumor necrosis factor alpha; IL-1: interleikin 1; IL-6: interleukin 6; CRP: C-reactive protein; Lysoz.: lysozyme; Ca: calcium; Mg: magnesium; P: phosphorus; Na: sodium; K: potassium; Cl: chloride; Uric. Ac.: uric acid; Bilir.: bilirubin; Tot. Pr.: total protein; Chol: cholesterol; Trigl.: triglycerides; CFU – colony forming units (total bacterial load); Colif.: coliform bacteria.

Supplementary Table 3. Age-related correlations in conventional, non-conventional, oxidative, immunological and bacteriological semen quality characteristics in senior boars.

	Age	Volume	Conc.	Motility	Memb. I.	Acros. I.	Apopt.	Necrot.	Mit. Act.	DNA Fr.	ROS	TAC	Prot. Ox.	LPO	Leukoc	TNFα	IL-1	IL-6	CRP	Lysoz.	Ca	Mg	P	Na	K	Cl	Urea	Uric Ac.	Bilir.	Tot. Pr.	Albumin	Chol.	Trigl.	CFU	Colif.
Age	1																																		
Volume	0.505**	1																																	
Conc.	0.320	0.206	1																																
Motility	-0.260	-0.384*	-0.364*	1																															
Memb. I.	-0.595**	-0.477*	-0.494*	0.635*	1																														
Acros. I.	-0.225	-0.454*	-0.316*	0.849**	0.726***	1																													
Apopt.	0.544**	0.305	0.905***	-0.555*	-0.737***	-0.493*	1																												
Necrot.	0.168	0.122	0.702**	-0.494*	-0.425*	-0.479*	0.667*	1																											
Mit. Act.	-0.312	-0.189	-0.302	0.845***	0.377*	0.639**	-0.472*	-0.530**	1																										
DNA Fr.	0.813***	-0.202	0.394*	-0.494*	-0.835***	-0.500**	-0.433*	0.607**	-0.686**	1																									
ROS	0.466*	0.221	0.521**	-0.590**	-0.663**	-0.651**	0.601**	0.790***	-0.442*	0.676**	1																								
TAC	-0.177	-0.191	-0.345*	0.472*	0.499*	0.741***	-0.528**	-0.773***	0.512*	-0.4928	-0.824**	1																							
Prot. Ox.	0.791***	-0.238	0.694**	-0.358*	-0.432*	-0.389*	0.688**	0.443*	-0.450*	0.443*	0.590**	-0.559**	1																						
LPO	0.570**	0.223	0.630**	-0.363*	-0.643**	-0.643**	0.704**	0.904***	0.540**	0.529*	0.890***	-0.964***	0.531*	1																					
Leukoc	0.577**	0.616**	0.452*	-0.449*	-0.498*	-0.365*	0.651**	0.329*	-0.478**	0.594*	0.657**	-0.433*	0.860**	0.648**	1																				
TNFα	0.460*	-0.241	0.252	-0.496*	-0.438*	-0.787**	0.507**	0.813*	-0.465*	0.618**	0.480*	0.780**	0.580**	0.790***	0.669**	1																			
IL-1	0.387*	0.123	0.500**	-0.546*	-0.585**	-0.729**	0.713***	0.492*	-0.682**	0.573*	0.544**	0.509**	0.531*	0.370*	0.545*	0.544**	1																		
IL-6	0.741**	-0.128	0.383*	-0.681**	-0.554**	-0.713**	0.622**	0.416*	-0.813***	0.879***	0.555**	0.489*	0.419*	0.411*	0.576*	0.857***	0.812***	1																	
CRP	0.189	0.122	0.357*	-0.440*	-0.654**	-0.503**	0.710**	0.339*	-0.378*	0.723**	0.394*	0.550**	0.385*	0.418*	0.550*	0.507**	0.541**	0.632**	1																
Lysoz.	-0.344*	-0.194	-0.440*	0.776**	0.440*	0.510**	-0.915***	-0.382*	0.407*	-0.471*	-0.439*	0.481*	-0.729*	-0.441*	-0.729***	-0.615**	-0.922***	-0.541**	-0.366*	1															
Ca	-0.530**	0.320*	-0.201	0.842**	0.217	0.377*	-0.214	-0.043	0.383*	-0.334*	-0.213	0.159	-0.232	-0.032	-0.263	-0.141	-0.139	-0.139	-0.012	0.424*	1														
Mg	-0.669**	-0.105	-0.213	0.578**	0.362*	0.353*	-0.223	-0.072	0.546*	-0.352*	-0.130	0.223	-0.187	-0.285	-0.386*	-0.183	-0.071	-0.135	-0.176	0.226	0.724***	1													
P	-0.722***	-0.202	-0.459*	0.116	0.067	0.117	-0.237	-0.230	0.143	-0.196	-0.258	0.238	-0.152	-0.154	-0.218	-0.066	-0.190	-0.143	-0.528	0.541**	0.405*	0.201	1												
Na	0.394*	0.267	0.161	0.273	0.114	0.261	-0.162	-0.198	0.208	-0.136	-0.128	0.076	-0.155	-0.140	-0.180	-0.671	-0.036	-0.046	-0.017	0.393*	-0.607**	-0.317	-0.103	1											
K	-0.656**	-0.159	-0.321*	0.413*	0.372*	0.160	-0.187	-0.195	0.017	-0.147	0.072	0.067	-0.119	-0.115	-0.277	-0.123	-0.102	-0.172	-0.081	0.256	-0.169	0.436	0.116	-0.676**	1										
Cl	0.241	0.201	0.343*	0.234	0.122	0.289	-0.144	-0.087	0.298	-0.159	0.250	0.043	-0.739	-0.131	-0.178	-0.075	-0.089	-0.066	-0.162	-0.181	-0.450*	-0.679**	-0.240	0.946***	-0.542*	1									
Urea	0.241	-0.188	0.174	-0.322	-0.022	-0.246	0.088	0.079	-0.144	0.055	0.244	-0.117	0.169	0.131	-0.099	0.038	0.125	-0.168	-0.057	-0.140	0.181	0.182	0.113	-0.271	0.022	-0.130	1								
Uric Ac.	0.067	-0.179	0.121	0.739**	0.303	0.365*	-0.298	-0.219	0.508**	-0.249	-0.449*	0.773**	-0.550**	-0.602**	-0.546*	-0.636**	-0.216	-0.184	-0.186	0.650**	0.215	0.222	0.593**	0.039	0.197	-0.136	0.010	1							
Bilir.	-0.581	0.112	0.225	-0.292	-0.196	-0.050	0.049	0.173	0.146	0.157	0.016	-0.065	-0.039	0.203	0.187	-0.183	0.124	0.025	0.199	0.225	0.192	0.239	-0.075	-0.169	0.095	-0.051	0.469*	-0.240	1						
Tot. Pr.	-0.795***	-0.265	-0.447*	0.488*	0.408*	0.552**	-0.516**	-0.511*	0.546**	-0.231	-0.113	0.392*	-0.680**	-0.270	-0.603**	-0.529*	-0.322*	-0.416*	-0.041	0.364*	0.619**	0.867**	0.627**	-0.182	0.228	-0.172	0.473*	-0.048	0.413*	1					
Albumin	-0.732**	-0.202	-0.351*	0.452*	0.451*	0.566**	-0.324*	-0.328*	0.585**	-0.356**	-0.294	0.463**	-0.685**	-0.118	-0.518*	-0.573*	-0.333*	-0.596**	-0.242	0.455*	0.748**	0.985***	0.720**	-0.130	0.434*	-0.190	0.599**	0.073	0.311	0.957***	1				
Chol.	0.267	0.198	0.380*	0.405*	0.423*	0.431*	-0.213	-0.150	0.537**	-0.304*	0.179	0.086	0.163	-0.471*	-0.313	-0.174	-0.104	-0.110	-0.012	0.254	0.148	-0.115	-0.185	0.194	-0.118	0.148	-0.205	-0.001	-0.413*	-0.184	-0.222	1			
Trigl.	-0.280	-0.064	0.360*	0.269	0.381*	0.393*	-0.259	0.145	0.364*	0.087	0.130	0.157	0.046	-0.444*	-0.226	-0.082	-0.279	-0.159	-0.177	0.107	0.183	-0.164	0.207	-0.112	-0.190	-0.125	0.254	-0.155	0.214	0.183	0.221	0.803**	1		
CFU	0.438*	0.124	0.510*	-0.978***	-0.512**	-0.578**	0.600*	0.559*	-0.788**	0.428*	0.682**	-0.463*	0.511**	0.541*	0.708**	0.720***	0.850**	0.520*	0.411*	-0.849***	-0.832***	0.685**	-0.617**	-0.641**	-0.573*	-0.546*	-0.534*	-0.640**	-0.667**	-0.558*	-0.476*	-0.568*	-0.283	1	
Colif.	0.369*	0.214	0.409*	-0.719***	-0.530**	-0.765**	0.607**	0.870*	-0.459*	0.435*	0.709**	-0.493*	0.918***	0.659**	0.959***	0.757***	0.925***	0.700**	0.573**	-0.608**	-0.422*	0.783***	-0.938***	-0.793***	-0.560*	-0.887**	-0.541*	-0.557**	-0.610**	-0.785**	-0.853***	-0.804**	-0.299	0.848***	1
	Age	Volume	Conc.	Motility	Memb. I.	Acros. I.	Apopt.	Necrot.	Mit. Act.	DNA Fr.	ROS	TAC	Prot. Ox.	LPO	Leukoc	TNFα	IL-1	IL-6	CRP	Lysoz.	Ca	Mg	P	Na	K	Cl	Urea	Uric Ac.	Bilir.	Tot. Pr.	Albumin	Chol.	Trigl.	CFU	Colif.

The interpretation of the results was based on the value of the Pearson's correlation coefficient: 0.111–0.333: weak correlation; 0.334–0.666: moderate correlation; 0.667–0.999: strong correlation. * – $p < 0.05$; ** – $p < 0.01$; *** – $p < 0.001$. Conc.: sperm concentration; Memb. I.: membrane intergity; Acros. I.: acrosome integrity; Mit. Act.: mitochondrial activity; DNA Fr.: sperm DNA fragmentation; ROS: reactive oxygen species; TAC: total antioxidant status; Prot. Ox.: protein oxidation (protein carbonyls); LPO – lipid peroxidation; Leukoc.: leukocyte levels; TNFα – tumor necrosis factor alpha; IL-1: interleikin 1; IL-6: interleukin 6; CRP: C-reactive protein; Lysoz.: lysozyme; Ca: calcium; Mg: magnesium; P: phosphorus; Na: sodium; K: potassium; Cl: chloride; Uric. Ac.: uric acid; Bilir.: bilirubin; Tot. Pr.: total protein; Chol: cholesterol; Trigl.: triglycerides; CFU – colony forming units (total bacterial load); Colif.: coliform bacteria.
